# Identifying feature relevance for transition from Questionable‐ to Low‐ AD using Interpretable‐AI

**DOI:** 10.1002/alz.084397

**Published:** 2025-01-09

**Authors:** Sai Sanhitha Beerapalli

**Affiliations:** ^1^ Centre for Brain Research, Indian Institute of Science, Bangalore, Karnataka India

## Abstract

**Background:**

In the face of increasing Mild Cognitive Impairment(MCI) and Dementia rates among aging populations, understanding the factors shaping the non‐normal cognitive decline is crucial. Leveraging the Clinical Dementia Rating (CDR) data, this study has a dual focus. (1) It utilizes CDR to aid early MCI diagnosis by investigating factors contributing to transition from Questionable‐ to Low‐ AD. (2) It undertakes a comparative analysis between Indian and Caucasian populations, acknowledging cultural influences on cognitive health.

**Method:**

SHAP analysis (Lundberg et al, 2017), a game‐theory based explainable‐AI technique, performed on classifier accurately classifying Questionable‐ and Low‐AD subjects of Indian population (LASI‐DAD study) and Caucasian population(ADNI‐CDR study) to identify relevance of features for transition from Questionable‐ to Low‐AD. In extension, scatter Index analysis of SHAP values help identify the distribution of influence along with the the pivotal features that played significant role in the classification.

**Result:**

Balanced datasets comprising 472 subjects of Caucasian population and 188 subjects of Indian population have been examined. SHAP analysis of logistic regression model with classification accuracy of 98%‐ADNI and 97%‐LASI yielded highest mean absolute value for Community domain (0.13) in ADNI data and Orientation domain (0.14) in LASI‐DAD data. Scatter Index of SHAP values resulted in scatter index deviation of 14.8% for Questionable‐AD and 26.8% for Low‐AD in Caucasian population; and 13.9% for Questionable‐AD and 13.6% for Low‐AD in Indian population. The analysis revealed that for ADNI data the distribution of feature importance is highly skewed to Community Affairs and for LASI data the feature importance is highly skewed to Orientation skills.

**Conclusion:**

Performance of individuals in Orientation domain is the most important feature affecting the transition from Questionable‐AD to Low‐AD in the Indian population, while Community affairs is the most important in Caucasian population. Worsening of the performance in those particular domains is the biggest key factor in conversion to Low MCI. Although CDR is heavily dependent on Memory scores, while analyzing the transition from Questionable‐AD to Low‐AD in particular, it is not the most important factor.

